# Most and least important attributes for domestic travel: A best-worst scaling approach

**DOI:** 10.3389/fpsyg.2022.987384

**Published:** 2022-09-20

**Authors:** Soyeun Olivia Lee, JooHyang Kim, Heesup Han

**Affiliations:** ^1^Faculty of Hospitality and Tourism Management, Macau University of Science and Technology, Taipa, Macau SAR, China; ^2^Department of Hotel and Airline Management, Hannam University, Daejeon, South Korea; ^3^College of Hospitality and Tourism Management, Sejong University, Seoul, South Korea

**Keywords:** COVID-19, domestic travel, travel selection attribute, best-worst scaling, relative importance

## Abstract

This study identifies the most important and least important selection attributes in Korean domestic travel during the COVID-19 pandemic. A total of 632 responses were used as the final analysis by conducting a survey of Koreans who have experienced travel in Korea since the outbreak of COVID-19. In order to explain tourists’ domestic travel selection preferences, best-worst scaling was used. As a result of the study, it was found that the destination environment is most important and is considered relatively important in the order of accommodation, major activities, expenditure, and crowdedness. On the other hand, length of stay, transport mode, travel time, and media exposure showed relatively low importance. In a situation where overseas travel is unstable due to the risk of infectious diseases, it is urgent to identify changes in domestic travel trends/factors that are important to tourists and respond to their needs and expectations. This study is academically expanded in that it not only bridges the research gap that previous studies have missed but also considers and ranks the importance of domestic travel factors at the same time.

## Introduction

The tourism industry is facing a major crisis as movement between countries is prohibited or limited due to the global spread of COVID-19 (Zenker and Kock, 2020; Çetin and Coşkuner, 2021; Yang et al., 2021a,b). The international tourism and hospitality industry has been severely hit by the COVID-19 pandemic (Kim et al., 2022), with international tourist arrivals down 74% year-over-year in 2020 (Çetin and Coşkuner, 2021). After the pandemic, the keyword ‘oversea travel’ has disappeared, and people around the world are waiting for its end. Although international travel is going through difficult times due to COVID-19, domestic tourism seems to have a good opportunity to develop (Brouder et al., 2020; Das and Tiwari, 2021). For example, according to recent Chinese tourism market statistics, domestic tourism revenue and people’s movement are increasing, which suggests a strong rebound in the Chinese tourism industry (Wen et al., 2020). In addition, Joo et al. (2021) emphasized that domestic tourism can serve as a strong substitute for overseas travel in a situation where overseas travel is unstable due to the risk of infectious diseases.

Meanwhile, COVID-19 has also significantly changed the types of tourism preferences and tourist behaviors (Kock et al., 2020; Lee et al., 2021; Yang et al., 2021a,b). Brouder (2020) stated that in relation to COVID-19, domestic tourists who enjoy traveling in an independent space or enjoying nature-friendly travel are increasing. Preference is a kind of attitude and plays a very important role when consumers make decisions. In marketing, Keller (2001) argued that when consumers have attributes and advantages to satisfy their needs, a preference for a brand arises and a positive attitude is formed. In this respect, preference, the degree to which a specific alternative is preferred among available alternatives, is one of the major factors influencing selection behavior (Dhar, 1997). Tourist preference is defined as the psychological tendency of visitors to depend on a specific tourist destination to select tourist destination (Tucker, 1967; Song et al., 2019).

Tourists are more likely to consider various options before making a choice and choose the most preferred tourism product/destination among them (Han et al., 2021; Kim et al., 2021). If a tourist destination is perceived as dangerous, it is less likely to be selected as a final destination (Han et al., 2019). Likewise, if the perceived risk is high, such as during COVID-19, it is expected that the preference for domestic travel will increase to reduce the uncertainty of overseas travel. Therefore, in the era of COVID-19, it is important to set up a strategy tailored to the trend that will emerge mainly in domestic travel and respond appropriately. Accordingly, in order to study the impact on the tourism industry and the changes in tourists’ tourism patterns through the COVID-19 crisis, it is necessary to know the changes in domestic travel preferences and trends. More specifically, this study aims to examine the relative importance of attributes for Korean domestic travel where the tourism market and form are completely changing due to COVID-19. Although many studies analyzing the selection attributes of domestic travel were conducted before COVID-19 (Bujosa et al., 2015; Fakfare et al., 2020; Campos-Soria et al., 2021), this analysis needs to be re-conducted (Toyama, 2021). This is because, when a special circumstance such as the era of new infectious diseases is studied, not only new selective attributes will emerge, but also existing attributes, such as accommodation and destination environment that are related to hygiene and infection, are highlighted (Pappas and Glyptou, 2021). Therefore, it is always necessary to identify the overall tourism selection attributes that are important to current tourists (Jang et al., 2021). In this respect, this study not only fills the research gap but also has a significant contribution to the existing literature.

To achieve the research objective, this study conducted a best-worst scaling (BWS)-based survey on domestic travel products under the assumption that each domestic travel selection attribute is a set of multiple choices in the same layer. BWS is a method in which a survey respondent is asked to repeatedly select the best and worst attributes for each set of choices. The method evaluates the priority among various attributes by analyzing the distance between the two attributes located on the measure of potential utility in the respondents’ minds. Because each respondent has a different importance and scale interval, it is not easy to strictly measure the respondent’s attitude toward each item with the existing preference evaluation method such as the Likert scale. However, since BWS creates a designed survey that allows respondents to select only one choice for each of the most important attributes, it is possible to measure preferences more clearly than the existing methods. The BWS approach reduces the possibility of bias by allowing respondents to respond more intuitively to the questionnaire (Goodman et al., 2005). BWS can be classified into three types according to the method of presenting attributes and levels. This study was conducted based on BWS case 1, which can compare and confirm conflicting attributes/factors in the same tier.

## Literature review

### Attributes of domestic travel and its importance

Mayo and Jarvis (1981) stated that travel is a set of choices and decisions made up of many attributes/factors, which include destination environment, accommodation, major activities, travel duration, expenditures, etc. The attributes of domestic travel selection have been confirmed through various studies: accommodations, crowdedness, environment, event/festival, expenditure, season, travel time, and transport mode, for the Australian domestic tourism destinations (Huybers, 2003; Pike, 2003; Massidda and Etzo, 2012). Similarly, Pike (2003) identified destination attributes that are important for New Zealand domestic travelers, and they can be narrowed down to accommodation, environment, destination activities, expenditure, travel time, locals, infrastructure, crowdedness, weather, and events. Based on the previous literature, a total of nine major determinants are decided in this study, which included accommodation, crowdedness, environment, expenditure, main activities at destination, media/SNS exposure, transportation mode, travel duration, and travel time. Table 1 shows a list of prior studies that referenced each selection attribute.

**Table 1:** Previous studies referring to attributes.

Attribute	Previous studies
Accommodation	Pike (2003), Grigolon et al. (2012)
Crowdedness	Tse et al. (2002), Huybers (2003), Kim and Stepchenkova (2015)
Environment	Huybers (2003), Pike (2003), Massidda and Etzo (2012)
Expenditure	Huybers (2003), Pike (2003), Massidda and Etzo (2012)
Main activities	Pike (2003), Massidda and Etzo (2012), Wen et al. (2020)
Media/ SNS Exposure	Karl et al. (2020), Majeed et al. (2020)
Transport mode	Huybers (2003)
Travel duration	Alegre and Pou (2006), Park et al. (2020), Wachyuni and Kusumaningrum (2020), Mirzaei et al. (2021)
Travel time	Oh et al. (2007), Grigolon et al. (2012), Romagosa (2020)

Five attributes - accommodation, environment, expenditure, major activities, and transportation mode - are related to domestic travel and have been mentioned both in previous studies and Korea National Travel Survey, so they were selected as major optional attributes in this study. The National Travel Survey is a report that provides basic data for policy establishment, research, and analysis on national tourism by comprehensively understanding the travel conditions of the Korean people. It is published by the Korea Culture and Tourism Institute every year. The remaining four attributes (crowdedness, travel duration, travel time, and media exposure) are not only basic components of travel but also attributes that have witnessed changes in tourists due to the COVID-19 pandemic in recent literature. Crowdedness is a measure of how busy tourist destinations are and is considered one of the most important factors in a pandemic situation. For example, Kock et al. (2020) found that psychological concepts of crowdedness had a significant impact on people’s perceived COVID-19 infectability. Crowdedness and safety measures also have been found to affect consumers’ choice of eating in or takeout at restaurants (Wang et al., 2021). Travel duration was selected as one of the attributes related to COVID-19 because many recent studies have reported that the length of travel is perceived as a risk to health, and therefore people prefer short-term trips (Wachyuni and Kusumaningrum, 2020; Baños-Pino et al., 2021; Mirzaei et al., 2021). Baños-Pino et al. (2021) investigated the impact of the COVID-19 pandemic on the length of stay of tourists based on data on visitors to Spain before and after the pandemic began. The study found evidence of a decrease in the length of stay by approximately 1.26 nights (23.8%). Travel time, which is the time taken from home to destination (Oh et al., 2007; Grigolon et al., 2012), was considered an important attribute of domestic tourism during the pandemic situation. Travel time is known as the cost paid for reaching a destination in the field of economics (Jain and Lyons, 2008). Li et al. (2021) and Romagosa (2020) emphasized that, under the epidemic situation, a lot of tourism demand is met close to home. In COVID-19, media exposure was also determined as an important attribute of this study, as tourists prefer relatively less-known places and destinations that have the least capacity and are not crowded (Karl et al., 2020). In general, a good reputation or fame of a tourist destination tends to lead to congestion (Tse et al., 2002).

### BWS method

BWS is an analytical technique that evaluates and prioritizes an individual’s relative importance with respect to various selection attributes constituting a specific research object, such as a product, service, or program. BWS began in 1987 when Louviere became interested in new information that could be obtained by asking additional questions about the ‘least preferred’ attribute, along with the traditional questionnaire that asked respondents to select the ‘most preferred’ attribute. At this time, the researcher repeatedly presents a selection set in which three or more selection attributes are variously combined to the respondent in the form of a questionnaire and has them select the most important or least important attribute in the questionnaire each time, one for each. This is a new and intuitive method of measuring consumer preference in the form of an extension of Thurstone (1927) pairwise comparison method, which compares two alternatives and selects only one more important alternative (Goodman et al., 2005). BWS has been proposed as a method to supplement the problems of quantitative methodologies such as rating scales and ranking scales. In a survey using a quantitative method, when the number of questions to be answered is large, questions are raised about the results of the survey because of the possibility that respondents will not continue to give sincere answers (Krosnick, 1999). Finn and Louviere (1992) pointed out the drawbacks of the rating scale such that it could be answered as similarly important several items because it was difficult for respondents to distinguish and evaluate different items. In addition, because it is often difficult to know the reliability and validity of the rating scale, BWS was proposed as a supplementary method. In the case of the rating method, since the importance of preference among respondents is different, ratings or rankings are not used in the same way, so each respondent can perceive a scale differently. Therefore, when bias occurs in survey results, there may be problems in that the result scores or grades are derived too similarly or interpretation is difficult (Goodman et al., 2005). This is because the importance of preference differs from person to person, so the interval of importance in the respondent’s mind is not the same. On the other hand, BWS can solve the problem of bias of a scale that assigns equal weight to all alternatives while considering the relativity between alternatives by allowing only one highest and one lowest alternative to be selected from a selected item. In particular, when the number of alternatives increases, the decision-making process of respondents is quick and simple. Based on these advantages, BWS has been utilized in various fields and has been mainly used for determining policy alternatives, designing marketing strategies, analyzing consumer preferences in health care/medicine, and food-related industries (Lagerkvist, 2013; Louviere et al., 2013; Massey et al., 2015; Mühlbacher et al., 2016). For example, Lagerkvist (2013) compared attribute importance rankings for beef labeling using BWS and direct ranking methods. Cohen (2009) used the BWS method to show the priorities of several attributes that consumers consider when choosing wines in retail stores. In the hospitality industry, Kim et al. (2019) used BWS to identify the most and least important selection attributes for consumers to choose luxury and economy hotels.

In this research, we analyze the relative importance of the attributes considered by domestic tourists as family members in domestic travel. BWS consists of multiple-choice sets with at least three items and is classified into object case (or Case 1), profile case (or Case 2), and multi-profile case (or Case 3) according to the complexity of the items (Louviere et al., 2015). BWS Case 1, which determines the priority of an attribute by measuring the relative importance of the attribute, is conducted.

## Materials and methods

### Study design

The domestic travel attributes to be measured in this study were derived based on the attributes selected as domestic travel attributes in previous studies and items used in Korea National Travel Survey. A total of nine attributes were selected, which included accommodation, crowdedness, duration, expenditure, environment, main activities at destination, media exposure, transportation mode, and travel time.

Accommodation is defined as a place where tourists sleep while traveling for tourism purposes and also refers to places equipped with facilities for the convenience of tourists and receiving a certain fee corresponding to their use (Ozdemir and Met, 2012). Destination environment refers to the setting of the place where the vacation takes place. Expenditure is expenses incurred from the time of travel, including transportation costs, accommodation costs, meals, and entrance fees to tourist attractions (Hong et al., 2005). The main activities are activities that tourists mainly plan and execute at their destinations. Transportation mode refers to the means of transportation in travel, and mainly includes cars, trains, and airplanes (Grigolon et al., 2012).

As an analysis method to achieve the purpose of the study, BWS, which can compare and confirm different attributes in a trade-off at the same level, was used. For the BWS survey, a selection set must be constructed so that the most important attribute and the least important attribute can be selected by comparing each conflicting attribute in the respondent’s mind (Flynn and Marley, 2014). At this time, if the number of attributes included in the selection set is different, respondents may be confused. In addition, if the questions are repeated, the total number of questions to be answered increases, which may hinder sincere and correct responses. Considering this, the selection set was constructed according to the balanced incomplete block design (BIBD), which is the most popular selection set construction method when performing the aggregation method. According to this approach, when multiple attributes in the selection set are divided into a small number of subsets, the selection set is constructed so that all selection attributes are included the same number of times, making it easier for respondents to make comparisons (Cohen, 2009). If we have many attributes to compare, which increases the total number of subsets and the total number of attributes that belong to the subset, BIBD can be used to adjust the number of each pair to compare (Raghavarao and Padgett, 2005). BIBD is based on the Latin square design, placing n attributes/levels in n rows and n columns.

The analysis was performed using the *R program*, and nine selection attributes were included a total of 3 times for every 12 generated subsets. Each subset was used as a BWS questionnaire item in this study, and Table 2 is one of a total of 12 BWS items used in this study.

**Table 2:** Example of best-worst scaling question from the survey.

Most important	Attribute for domestic travel	Least important
1	Travel duration	1
2	Travel time	2
3	Accommodation	3

### Data collection

In the tourism market, families are the group that occupies the largest consumption segment in the consumption market (Srnec et al., 2016), and only small-scale trips such as family trips have been made intermittently after COVID-19 (Chebli and Said, 2020; Jiang and Wen, 2020). In order to provide specific and accurate implications based on the results of the study, the largest consumption group, family members (married people in their 30 s and 40 s), was the subject of the questionnaire. First of all, thirty people in their 30 s and 40 s were recruited, and a preliminary survey was conducted to confirm and supplement whether the respondents could properly understand the textual items and whether there were any difficulties in responding. The final questionnaire was completed after review by two tourism experts (one professor and one travel agency representative). In February 2021, this survey was conducted for family members in their 30 s and 40 s who had domestic travel experience after COVID-19, and a total of 632 copies were used as final analysis data.

### Data analysis

It is impractical to design a survey in such a way that all factors are compared with each other to prioritize domestic travel attributes. Therefore, a BIBD, which is designed to be no correlation and interaction between factors, was used. Another advantage of using a BIBD design is that it can significantly reduce the number of selection sets to evaluate while maintaining a balanced and joint shape of items throughout the set (Green, 1974). A counting approach was used for the analysis method of the questionnaire data. The counting approach is based on the frequency at which purchasing factor is selected as the most important and least important purchasing factor from a set of 12 choices by all respondents N (632 people). The priority and relative importance of domestic travel attributes can be grasped using the score derived from the following equation (Aizaki et al., 2014).

$BW_{i}=B_{i}-W_{i}$ Equation (1)

$std.BW_{i}=\frac{BW_{i}}{N_{r}\;}$ Equation (2)

$sqrt.BW_{i}=\sqrt{\frac{B_{i}}{W_{r}\;}}$ Equation (3)

$std.sqrt.BW_{i}=\frac{std.BW_{i}}{\max.std.BW_{i}\;}$ Equation (4)

In Equation (1), *B_i_* denotes the frequency of responding to attribute *i* as the most important domestic travel attribute, *W_i_* denotes the frequency of responding to attribute *i* as the least important domestic travel attribute, and *BW_i_* represents the difference in frequency between *B_i_* and *W_i_*. In Equation (2), N is the number of respondents and *r* is the number of times attribute *i* was included in the 12 selection sets (all domestic travel attributes were included in the selection set three times, so *r* becomes three). *std.BW_i_* means the difference between the standardized *B*_i_ and *W*_i_ and is used to determine the order of priority among the nine attributes. *sqrt.BW_i_* in Equation (3) is the square root of the ratio of *B_i_* and *W_i_*. *Max.std.BW_i_* of Equation (4) is the maximum value of *sqrt.BW_i_*. The *std.sqrt.BW_i_* indicates the relative importance between items. For example, if *std.sqrt.BW_i_* is 0.6 and *std.sqrt.BW_j_* is 0.19, then the resultant scale means that item *i* is about three times as important as item *j* (0.6/0.19 ≈ 3.2).

## Results

### Sample overview

The respondents included slightly more females (50.2%) than males (49.8%). The age distribution in their 30 s and 40 s is even at 49.4 and 50.6%, respectively. More than half of the sample possessed a university degree (56.2%) or graduate degree (9.1%). The average monthly family income was approximately USD 4,000 (USD 1 = 1,200 Korean Won) and about half of the respondents (49.4%) of the sample are office workers. Approximately 23% of respondents do not have children and having one or two children is the dominant (72.1%) response.

### Analysis result of BWS

Table 3 is the result derived by the counting approach. B is the frequency with which respondents answered that the most important domestic travel attribute, and W is the frequency with which they answered that it is the least important. BW is the difference in the frequency of the most important/least domestic travel attribute as a result of Equation (1). *std.BW*, *sqrt.BW*, and *std.sqrt.BW* are the results of equations (2), (3) and (4), respectively. The score of *std.BW* is used as an index indicating the priority among domestic travel selection attributes. If *sqrt.BW* = 1, then the number of times that respondents selected the attribute as most important and the number of times that it was not important were the same. If *sqrt.BW* > 1, then the number of times that respondents selected the attribute as most important is greater than the number of times that respondents selected the attribute as least important. On the other hand, if *sqrt.BW* < 1, then the number of times that respondents chose the attribute as least important was greater than the number of times those respondents selected the attribute as most important. In *std.sqrt.BW*, the first-priority attribute has a value of 1 and other factors have a value less than 1. This is an index comparing the relative importance between domestic travel attributes.

**Table 3:** Aggregated best-worst scores.

Attribute	*B*	*W*	*BW*	*std.BW*	*sqrt.BW*	*std.sqrt.BW*
Environment	1,580	305	1,275	0.540	2.276	1.000
Accommodation	1,278	458	820	0.324	1.67	0.734
Main Activities	970	667	303	0.119	1.206	0.530
Expenditure	883	629	254	0.100	1.185	0.521
Crowdedness	853	846	7	0.002	1.004	0.441
Duration	579	988	−409	−0.161	0.766	0.336
Transport mode	563	1,091	−528	−0.208	0.718	0.316
Travel time	476	1,078	−602	−0.238	0.664	0.292
Media/SNS exposure	402	1,522	−1,120	−0.443	0.514	0.226

As a result of calculating the importance of each attribute using the *sqrt.BW* value to achieve the research purpose, it was found that domestic travelers leaving for domestic travel after the onset of COVID-19 consider environment (0.54) as the most important, and accommodation (0.324), main activities (0.119), expenditure (0.100), crowdedness (0.002), were found to be considered relatively important in that order. In contrast, duration (−0.161), transport mode (−0.208), travel time (−0.238), and media exposure (−0.443) showed relatively low importance.

## Discussion

Most previous studies on COVID-19 and the tourism industry have focused on the analysis of the impact of the epidemic on the tourism industry or the recovery plan after the end of the epidemic (Brouder, 2020; Wen et al., 2020; Bae and Chang, 2021). Additionally, studies related to COVID-19 generally focus on exploring the impact this pandemic has on one specific travel factor, such as the tourism environment (Buckley and Westaway, 2020; Avraham, 2021; Çetin and Coşkuner, 2021), accommodation (Jiang and Wen, 2020; Atadil and Lu, 2021; Çetin and Coşkuner, 2021; Jang et al., 2021; Pavlatos et al., 2021), travel duration (Wachyuni and Kusumaningrum, 2020; Baños-Pino et al., 2021; Mirzaei et al., 2021), travel time (Romagosa, 2020; Li et al., 2021), and transport mode (Wen et al., 2020; Aparicio et al., 2021). In that respect, this study not only fills the research gap that previous studies have missed but also makes a great contribution academically in that it considers and ranks the importance of these factors constituting domestic travel at the same time. Of the nine attributes, most of them except media exposure have exclusively dealt with the impact of COVID-19 on this travel factor in previous studies. Hence, the attributes used in this study were individually verified empirically, and the analysis results supported the previous studies. However, in the case of media exposure, despite extensive discussion in the scientific literature about how media can influence people’s health-related emotions and behaviors (Scopelliti et al., 2021), the impact of media communications about COVID-19 on tourism behavior has been less explored. Reflecting this, media exposure was found to be the least important attribute among the nine domestic travel components. Now, we will take a look at the priorities according to the analysis results.

The study result reported that the environment is the most influential domestic travel determinant, which is also supported by many contemporary empirical studies (Buckley and Westaway, 2020; Kusumaningrum and Wachyuni, 2020; Li et al., 2020). Li et al. (2020) outlined the transformation in tourist behavior patterns since the COVID-19 outbreak and confirmed that the environment is an important factor in the COVID-19 situation. Kusumaningrum and Wachyuni (2020) also investigated the trends in travel change after the COVID-19 pandemic, with the majority of respondents reporting nature tourism as their most preferred choice. This behavior is believed to ensure that people traveling to urban areas where there is a relatively high risk of infection seek safe to travel in order to ensure safety even during domestic tourism. In other words, if possible, the phenomenon of leaving for travel activities in nature with less interpersonal contact appears (Freeman and Eykelbosh, 2020; Seraphin and Dosquet, 2020). Academically, the perception of anxiety about psychological and social risks according to the attention recovery theory is the same in that it acts as a catalyst to leave the natural environment (Kaplan and Kaplan, 1989). Consistency with this theory, Buckley and Westaway (2020) confirmed that walking in bountiful nature is helpful for the psychological treatment of women during COVID-19. As such, nature experience not only reduces various negative emotions such as stress, anxiety, depression, and boredom (Bratman et al., 2015) but also exposure to the natural environment has been shown to increase well-being and quality of life (Seraphin and Dosquet, 2020). Meanwhile, tourism demand is changing as tourists concentrate on tourist destinations with proven safety and not crowded with people. The World Travel and Tourism Council (WTTC), together with WHO and national quarantine authorities, has established a global protocol for ‘safe travel,’ issuing a safety certificate called ‘Safe Travels by WTTC’ to tourist destinations that comply with this (WTTC, 2020). Accordingly, government ministries in each country will have to work hard to acquire certificates to attract domestic and foreign tourists who will be revived in the future by guiding the regulations to tourist destinations across the country.

The accommodation was selected as the second important attribute. This study finding supports the results of recent previous studies that cleanliness should be guaranteed during travel as interest in hygiene management increases after COVID-19 (Gaur et al., 2021). According to the 2020 Expedia survey of 1,000 Americans, millennial parents had the highest rate of the travel keyword ‘clean’ since COVID-19 began (Expedia, 2020). Atkinson (1988) also cited cleanliness as the most important consideration for tourists when choosing specific accommodations. Besides, when choosing a travel destination or accommodation after COVID-19, consumers are more concerned about reliable hygiene management (Shin and Kang, 2020; Atadil and Lu, 2021). In line with this, the global tourism industry tends to introduce a certification system that proves the hygiene and safety of facilities and emphasizes the importance of disclosing information related to hygiene management in attracting customers (Çetin and Coşkuner, 2021; Pavlatos et al., 2021). For example, Pavlatos et al., (2021) study of the Greek hotel industry emphasized marketing aimed at convincing potential customers that it provides maximum safety to manage the pandemic situation. This paper also argued that the industry should aim to change operations and invest in new technologies such as electronic check-in/check-out and infrared thermal checks to ensure the hygiene and safety of hotel staff and guests. In this state of affairs, a hotel with strict hygiene management seems more advantageous in attracting guests than shared accommodation (Atadil and Lu, 2021).

As the third important attribute of domestic travel, main activities were selected. One of the biggest changes brought about by COVID-19 in daily life is the spread of the ‘untact’ culture (Bae and Chang, 2021). ‘Untact’ is a new word with the negative prefix ‘un’ added to the word ‘contact’ and refers to behavioral tendencies that minimize direct contact among Korean people (Bae and Chang, 2021). As a result of this, the tendency to prefer less face-to-face contact in tourism behavior has increased, and interest in safety in selecting tourism activities is greater than ever before (Freeman and Eykelbosh, 2020; Bae and Chang, 2021). Social distancing is one of the types of infectious disease management that means physical distancing or safe distancing. In Korea, as social distancing campaigns were implemented, measures have been also taken to restrict the operation of some facilities and industries, such as fitness clubs and concert halls, which have a high risk of infection. Therefore, the tendency to avoid confined places strengthened. Because major exhibitions and conventions were canceled, cultural activities such as museum tours and exhibition participation were severely restricted (Liu et al., 2021). In addition, as unnecessary gatherings and outings decreased, visits to entertainment facilities (casinos, clubs, amusement parks, etc.) and offline shopping activities were also reduced. Furthermore, due to measures taken to prevent the spread of COVID-19, such as closing multi-use facilities and canceling local festivals, citizens have lost the opportunity to sufficiently satisfy their desire for leisure and cultural life (Ratten, 2020). In this light, activities to heal the body and mind exhausted from COVID-19 with relaxation at travel destinations and natural scenery are growing more and more (Buckley and Westaway, 2020; Seraphin and Dosquet, 2020). Lee and Han (2022) also reported that gourmet and entertaining tours decreased compared to before the onset of COVID-19, while preference for relaxation and ecotourism increased significantly. Hence, tourism officials preparing for the post-COVID era should give priority to developing outdoor spaces such as parks or national parks rather than indoor spaces.

Expenditure was listed as the fourth important determinant of domestic travel. After the financial crisis in 2007, overseas travel demand in 2009 decreased significantly in the US, Australia, Canada, Japan, and EU-15 countries. As such, the economic crisis makes lives less prosperous and leads to austerity consumption (Smeral, 2010). An epidemic such as COVID-19 is also a disaster that adversely affects the tourism sector, just like the financial crisis (Ritchie and Jiang, 2019). In such a financially difficult situation, travel, which is not a necessity, has become an economic burden (Eugenio-Martin and Campos-Soria, 2014). Therefore, it is natural for individual consumption to shrink, as economic activities in all fields around the world contract due to the prolonged pandemic (Pham et al., 2021). On the other hand, as personalization and miniaturization expand, the preference for tourism activities with increasing family and individual travel is changing (Jiang and Wen, 2020; Wen et al., 2020). As such, as the number of travel products for only a small number of people increases, the perception of travel expenses is changing. For example, prices were lowered by forming economies of scale for many tourists in the past, but now the market for products that raise prices by involving fewer people is gradually expanding (Korea Culture and Tourism Institute, 2020).

The fifth attribute that appears to be considered relatively important is crowdedness. Non-face-to-face culture is spreading due to anxiety about contagion and the implementation of social distancing, leading to various tourism changes (Çetin and Coşkuner, 2021; Pavlatos et al., 2021). This suggests the need for changes in travel space, such as dispersion of tourism seasons and reduction in the density of tourist destinations, as the tendency to avoid enclosed and dense environments become stronger after COVID-19. The spread of the non-face-to-face consumption culture will also affect the timing of travel demand, which is expected to increase the demand for travel during the off-season when people travel relatively less (Atadil and Lu, 2021). These changes may contribute to bringing about the effect of time dispersion of travel demand. Expedia made a similar forecast, and it was expected that small and medium-sized cities with relatively small populations would be attracting attention as travel destinations, considering the population density of the area when deciding on a destination due to the impact of COVID-19 (Expedia, 2020). The fact that tourists’ interest in small-town areas has increased is very encouraging for the area, so the time has come for regional-led tourism to pay attention. In addition, a research review including an academic survey conducted in over 20 countries by Rogerson and Rogerson (2021) highlighted the contactless economy and untact tourism. A new paradigm based on social distancing has created a contactless economy era in all tourism consumption, including self-service counters, unattended kiosk check-in at hotels and golf clubs, and in-room dining. As this paradigm has emerged and rapidly grown in recent years, it is urgent to prepare countermeasures so that all age groups can accept it without difficulty.

The sixth most important attribute among the nine items was the travel period when considering the attributes of domestic travel. Travelers are aware of the seriousness of COVID-19 and change their itinerary to prevent infection (Wen et al., 2005; Wachyuni and Kusumaningrum, 2020; Baños-Pino et al., 2021; Mirzaei et al., 2021), but the length of stay does not appear to be a very important attribute as it is changeable. For example, changing a family trip from an amusement park to a national park seems big, but reducing the travel period from 2 nights to 1 night is not as big of a change as changing the destination.

The seventh most important attribute is transport mode. With the frequency of travel reduced due to COVID-19, people have become more reluctant to use public transportation such as airplanes and trains (Wen et al., 2020). Additionally, since it is family travel, the use of private cars has increased. Therefore, transportation appears to be a relatively less important attribute. Even if it is not limited to family travel, in fact, according to the results of the survey on national travel in Korea in the first quarter of 2021, travel by private car was 85.7%, which was much higher than 76.3% in the first quarter of 2019 before the outbreak of COVID-19. In the special circumstances of the epidemic, it is expected that tourists will be able to avoid group tours as they are reluctant to use public transport. However, it will not be possible to only travel on a small scale or use a private car either. Reflecting this special situation, public transportation will have to pay close attention to infection prevention, such as wearing a mask and providing hand sanitizer in vehicles.

The second least important attribute in determining domestic travel is travel time. Travel time is a characteristic that has a little direct relationship with infectious diseases. Nonetheless, current research reports that the COVID-19 pandemic has affected the proximity of destinations (Chebli and Said, 2020; Romagosa, 2020). It is said that people prefer short-distance travel in order to reduce the psychological burden when faced with certain problems or unexpected situations during travel (Li et al., 2021). According to the travel trends for 2021 announced by Airbnb (2020), the number of reservations for short-haul (50 miles or less) and medium-distance (50–500 miles) travelers has increased due to COVID-19. In addition, 25% of the respondents answered that they are more interested in areas close to their homes, so it can be expected that the travel time will be shorter. Travel to shorter travel distances has been reported in many countries, including France (Lebrun et al., 2021), South Korea (Lee and Han, 2022), and Spain (Donaire et al., 2021; Duro et al., 2021). Based on this evidence, it would be good for tourism industry officials to launch travel products focusing on short-distance travel in the post-COVID era.

The last attribute considered relatively important is media exposure. Many studies have predicted that COVID-19 will increase the tendency to favor visiting relatively less-known tourist destinations (Chebli and Said, 2020; Karl et al., 2020). In this light, the media exposure attribute was also expected to rank as an important attribute, but it appeared as the least important attribute because tourist destinations with high media exposure will have relatively large numbers of tourists, which people do not want. As the most insignificant result of domestic travel attribute (media exposure) was reflected in the actual literature, there are few prior studies on media exposure and travel intentions due to COVID-19 (Seyfi et al., 2021).

## Conclusion and implications

Domestic tourism has always existed, but it has not been able to achieve great development because it is insufficient to meet the sophisticated demand of tourists from overseas tourism. Meanwhile, the COVID-19 outbreak has created a substitution effect between international and domestic tourism flows (Brouder et al., 2020; Das and Tiwari, 2021). As such, now the demand for domestic tourism has increased, and the selection attribute of domestic tourist destinations has become very important (Karl et al., 2020). As a result of the study analysis, it was revealed that the most important attribute that respondents considered when deciding on domestic travel was the destination environment, and it was found that accommodation, main activities, expenditure, and crowdedness were considered important in that order. On the other hand, travel duration, transport mode, travel time, and media exposure were derived as attributes with relatively less importance. This study presented basic data for establishing tourism policies and marketing strategies for tourism industry stakeholders by analyzing the relative importance of domestic travel attributes using the BWS analysis technique. Therefore, the tourism industry stakeholders will be able to respond effectively according to the priorities suggested by the results of this study.

In the midst of the crisis in the tourism industry after COVID-19, it is necessary to actively respond to the following changes in tourism patterns. Based on the results of this study, the following practical implications were drawn. First, the destination environment was selected as the most important factor among the nine properties related to domestic tourism. According to a recent study, people prefer to travel to enjoy outdoor activities with less contact with other people, even in the tourist environment. Therefore, Korean industry officials should support and foster nature-friendly travel destinations that value safety and health. In order to respond to this, it is necessary to expand and select COVID-free safe tourism facilities and tourist destinations that are currently promoted in Korea and provide relevant information. In addition, it is necessary to discover and develop eco-tourism sites where people can enjoy nature more intensively. Second, accommodation officials should not only actively enforce their own infectious disease prevention efforts, but also promote this to customers. It is costly and time-consuming to implement the correct quarantine rules and guide tourists to take precautions to keep them in mind. However, this is important as it conveys trust to tourists that it is safe. First-class franchise hotels have been using a marketing strategy to reorganize and promote hygiene protocols for each brand. For example, Marriott Hotels are operating with enhanced hygiene and cleanliness protocols under the name of ‘Global Cleanliness Council’, Hilton Hotels ‘Clean Stay’, and Hyatt Hotels ‘Global Care & Cleanliness Commitment’ (GCCC). Although the name of the cleanliness program is different, it is a cleanliness and quarantine strengthening program developed to meet the changing expectations of customers during the COVID-19 pandemic. As such, it will be necessary to introduce such a cleanliness program in small hotels and tourist destinations other than large brand hotels. As main activities are selected as the third important attribute, the development of well-being and healing tourism products centered on outdoor activities related to the first important attribute (destination environment) should be expanded. Many previous studies have emphasized the importance and growth potential of wellness tourism, which seeks to heal the body and mind exhausted from COVID-19, promote health and improve quality of life (Buckley and Westaway, 2020; Chebli and Said, 2020; Freeman and Eykelbosh, 2020). In other words, wellness tourism can be said to mean enjoying spas, recreation, beauty, and health care that pursue tourism for the purpose of health and healing. Recognizing that the luxury market is growing even in the midst of the rise of the isolation economy (Pang et al., 2021), product planning in the direction of value consumption for oneself even in a crisis situation should be carried out.

This study conducted an empirical study based on previous research to analyze the attributes that domestic tourists who have traveled in Korea during the COVID-19 period consider when traveling. The following points are thought to be more helpful to academia and the tourism industry if additional research is carried out in the future. First, it is expected that more reliable and robust research results can be obtained if the attributes mainly mentioned in search engines are derived using big data analysis techniques and used for research. Second, a study comparing the importance of the selected attributes of domestic and foreign tourists by examining the selection attributes of foreign tourists traveling to Korea would be able to add diverse research results. Lastly, although this study utilized BWS1, which presents the best and worst attributes, BWS can be classified into three types according to the method of presenting attributes and levels. A follow-up study using BWS 2, which examines the most important (best) and lowest (worst) levels according to each attribute, would provide more specific implications for the tourism industry.

## Data availability statement

The raw data supporting the conclusions of this article will be made available by the authors, without undue reservation.

## Ethics statement

Ethical review and approval was not required for the study on human participants in accordance with the local legislation and institutional requirements. Written informed consent from the participants was not required to participate in this study in accordance with the national legislation and the institutional requirements.

## Author contributions

HH: survey questionnaire designing and editing. SL: data collecting, analysis, and drafting. JK: writing and editing. All authors contributed to the article and approved the submitted version.

## Conflict of interest

The authors declare that the research was conducted in the absence of any commercial or financial relationships that could be construed as a potential conflict of interest.

## Publisher’s note

All claims expressed in this article are solely those of the authors and do not necessarily represent those of their affiliated organizations, or those of the publisher, the editors and the reviewers. Any product that may be evaluated in this article, or claim that may be made by its manufacturer, is not guaranteed or endorsed by the publisher.
